# Host Cell S Phase Restricts *Legionella pneumophila* Intracellular Replication by Destabilizing the Membrane-Bound Replication Compartment

**DOI:** 10.1128/mBio.02345-16

**Published:** 2017-08-22

**Authors:** Dennise A. de Jesús-Díaz, Connor Murphy, Asaf Sol, Marion Dorer, Ralph R. Isberg

**Affiliations:** a Graduate Program in Molecular Microbiology, Sackler School of Graduate Biomedical Sciences, Tufts University School of Medicine, Boston, Massachusetts, USA; b Department of Molecular Biology and Microbiology, Tufts University School of Medicine, Boston, Massachusetts, USA; c Howard Hughes Medical Institute, Tufts University School of Medicine, Boston, Massachusetts, USA; University of Washington

**Keywords:** *Drosophila*, *Legionella pneumophila*, RNA interference, cell cycle, intracellular bacteria, secretion systems, vacuoles

## Abstract

*Legionella pneumophila* grows within cells ranging from environmental amoebae to human macrophages. In spite of this conserved strategy of pathogenesis, identification of host factors that restrict *L. pneumophila* intracellular replication has not been extended outside components of the mammalian innate immune response. We performed a double-stranded RNA (dsRNA) screen against more than 50% of the *Drosophila melanogaster* annotated open reading frames (ORFs) to identify host cell factors that restrict *L. pneumophila*. The majority of analyzed dsRNAs that stimulated *L. pneumophila* intracellular replication were directed against host proteins involved in protein synthesis or cell cycle control. Consistent with disruption of the cell cycle stimulating intracellular replication, proteins involved in translation initiation also resulted in G_1_ arrest. Stimulation of replication was dependent on the stage of cell cycle arrest, as dsRNAs causing arrest during S phase had an inhibitory effect on intracellular replication. The inhibitory effects of S phase arrest could be recapitulated in a human cell line, indicating that cell cycle control of *L. pneumophila* replication is evolutionarily conserved. Synchronized HeLa cell populations in S phase and challenged with *L. pneumophila* failed to progress through the cell cycle and were depressed for supporting intracellular replication. Poor bacterial replication in S phase was associated with loss of the vacuole membrane barrier, resulting in exposure of bacteria to the cytosol and their eventual degradation. These results are consistent with the model that S phase is inhibitory for *L. pneumophila* intracellular survival as a consequence of failure to maintain the integrity of the membrane surrounding intracellular bacteria.

## IMPORTANCE

*Legionella pneumophila* has the ability to replicate within human macrophages and amoebal hosts. Here, we report that the host cell cycle influences *L. pneumophila* intracellular replication. Our data demonstrate that the G_1_ and G_2_/M phases of the host cell cycle are permissive for bacterial replication, while S phase is toxic for the bacterium. *L. pneumophila* replicates poorly within host cells present in S phase. The inability of *L. pneumophila* to replicate relies on its failure to control the integrity of its vacuole, leading to cytosolic exposure of the bacteria and eventual degradation. The data presented here argue that growth-arrested host cells that are encountered by *L. pneumophila* in either the environment or within human hosts are ideal targets for intracellular replication because their transit through S phase is blocked.

## INTRODUCTION

Legionnaire’s disease is an atypical pneumonia caused by inhalation of aerosolized waters contaminated with the bacterium *Legionella pneumophila* (1). Pneumonic disease in humans is initiated after aspiration of contaminated aerosols and engulfment of the bacterium by alveolar macrophages (2), while in the environment, *L. pneumophila* can be found within an assortment of freshwater amoebal species (3). In all cell types, the ability of *L. pneumophila* to replicate and cause disease is dependent on the presence of the Icm/Dot type IV secretion system (T4SS) that allows construction of a *Legionella*-containing vacuole (LCV) associated with membranes from the endoplasmic reticulum (ER) (4). Each *L. pneumophila* strain encoding the T4SS is predicted to support the translocation of around 300 bacterial proteins into the host cytosol to modulate and subvert host functions (5). These include proteins that hijack host vesicle trafficking functions, interfere with the function of antimicrobial compartments, and protect the bacterium from host innate immune cytosolic sensing systems (6). Biochemical studies have identified translocated bacterial proteins that control the activity of a variety of host Rab GTPases (7–9), actin (10), sorting nexins (11), ubiquitin (12), and protein synthesis machinery (13).

As indicated from these biochemical studies, most of our knowledge regarding how *L. pneumophila* is able to replicate inside host cells has been focused on the activities of the T4SS-translocated proteins. It is believed that the combination of these activities controls formation and trafficking of the LCV within the cell (6). Other functions, however, exist that are involved in allowing the bacterium to avoid immune detection. Bacterial mutants lacking the SdhA protein, or which lack both LidA and WipB proteins, are defective for maintaining a protective niche that allows the bacterium to hide from cytoplasmic innate immune responses (14, 15). The inability to maintain an intact vacuole in this fashion results in host cell defenses being activated with consequent degradation of the bacterium, presumably through the exposure of bacterial lipopolysaccharide to the host cell cytoplasm (16). However, little is known about host pathways that interfere with intracellular replication of this pathogen that are not components of the host innate immune detection system.

Protein synthesis inhibition by *L. pneumophila* has emerged as a central feature of the infection process, but the role that this tactic plays in modulating intracellular replication is poorly understood (13, 17, 18). Protein synthesis inhibition in mammalian cells in response to *L. pneumophila* challenge appears to occur at two levels. First, up to seven different Icm/Dot-translocated proteins have been shown to interfere with host translation, many of which appear to target translation elongation (19). In mammalian cells, translation inhibition occurs at a second level, as a result of a host response to *L. pneumophila* challenge. In this case, degradation of the host mTOR protein in response to infection results in a block in translation initiation (20), and this strategy appears to be the primary mechanism of protein synthesis inhibition several hours after initial bacterial uptake (21). The consequences of each of these strategies on intracellular replication are unclear. Bacterial mutants that are defective for inhibiting translation elongation show no replication defect in mouse bone marrow-derived macrophages but have lowered intracellular growth in amoebae for unknown reasons (18), perhaps because the inhibitors block the host unfolded protein response (21, 22) a known form of innate immune protection in simple eukaryotes (23–25). Inhibition of translation initiation, in particular, appears to bias mammalian cells toward producing proinflammatory cytokines, conceivably allowing the immune response to contain bacterial replication (20).

To identify host factors necessary for optimal intracellular replication of *L. pneumophila*, we performed a large-scale unbiased screen in *Drosophila melanogaster* cells to identify host factors whose absence stimulates *L. pneumophila* intracellular replication, building on our previous work using secretory component-directed double-stranded RNAs (dsRNAs) (26). We show here that within dividing cells, inhibition of translation initiation and cell cycle blockade in G_1_ and G_2_/M stimulates intracellular replication by preventing progress through S phase and supporting maintenance of LCV integrity. These results are supported by recent work that shows that entry of *L. pneumophila* into *Acanthamoeba castellanii* results in blockage of proliferation and DNA synthesis within this host cell (27).

## RESULTS

### A high-throughput assay for identification of host components that modulate *L. pneumophila* intracellular replication.

**FIG 1:**
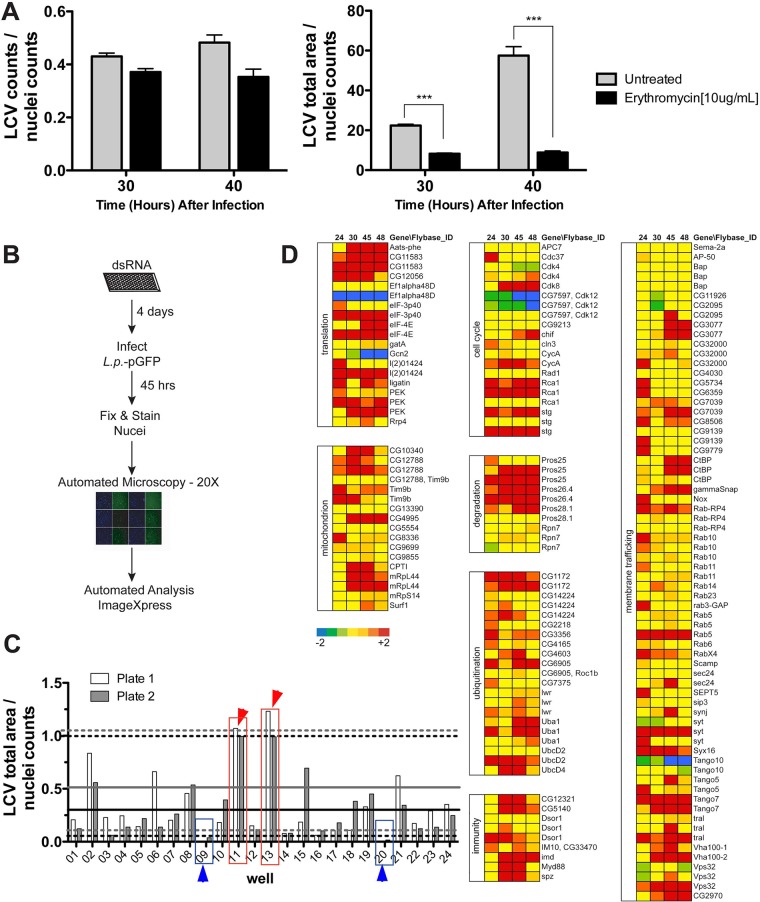
dsRNA screen in *D. melanogaster* cells to identify host factors involved in restriction of *L. pneumophila* intracellular growth. (A) The screening strategy allows detection of bacteria with altered intracellular replication. *Drosophila* cells were challenged with the Lp01 strain at the noted time points in absence or presence of erythromycin, added at 19 h postinfection. The total number of *Legionella* vacuoles was counted by automated microscopy (right panel), and the total LCV area (left panel) was calculated at 30 and 40 hpi and normalized to cell nuclei (Materials and Methods). (B) Flow chart representation of the dsRNA interference screen strategy used in *D. melanogaster* cells to identify host factors that modulate *L. pneumophila* intracellular growth. (C) Strategy for identification of hits with altered levels of bacterial growth during the screen. Phenotypes of interest due to increased levels of *L. pneumophila* growth are shown in red; phenotypes of interest due to reduced bacterial growth are shown in blue. Upper dashed lines represent the value for *Z* = 2 above the mean for each plate. Lower dash lines represent the value for *Z* = 1.5 below the mean for each plate. Solid lines represent the mean for each plate. Black lines, plate 1; gray lines, plate 2. (D) Candidate dsRNAs that result in enhanced *L. pneumophila* growth. In the secondary screen, Lp01 *lux*^*+*^ was introduced onto *Drosophila* cell monolayers, and luminescence was measured at the indicated times (hours) after uptake as a readout for growth. The color scale (displayed below the left column) represents SD from the mean replication levels obtained in an untreated control plate. Genes were categorized based on their function or host cell process.

To identify host factors that modulate *L. pneumophila* intracellular replication, we developed a high-throughput, whole-genome dsRNA interference screen strategy. To determine if we could detect subtle differences in *L. pneumophila* vacuole growth using a microscopy-based approach, we tested if differences in the *Legionella*-containing vacuole (LCV) size could be identified. Green fluorescent protein-positive (GFP^+^) *L. pneumophila* cells were incubated with *D. melanogaster* Kc167 cells at 25°C, and bacterial replication was halted after a few bacterial divisions by addition of erythromycin 19 h after challenge. The vacuole size of the antibiotic-inhibited cells was compared to that of cells that were incubated in the absence of the antibiotic. No differences in the total number of LCVs in the presence or absence of antibiotic were observed at any of the times tested (Fig. 1A, left panel). In cells treated with erythromycin, there was a significant decrease in the LCV size relative to untreated controls by 30 or 40 h of infection (Fig. 1A, right panel, 11 to 21 h post-erythromycin addition).

In order to be able to select size differences in a high-throughput approach, we determined the *Z* score that would be expected for a typical mutant by using a dsRNA that interferes with *L. pneumophila* intracellular replication (26) and then performing replicates (*n =* 23) of this dsRNA compared to a large number of replicates of untreated controls (*n =* 300 wells). Kc167 cells were treated with dsRNA interference (dsRNAi) against *ufd1* for 4 days and then challenged with *L. pneumophila* GFP^+^ cells. The probability of losing this mutant based on a particular *Z* score was then determined (see Table S1 in the supplemental material). Based on this evaluation, the lowest probability of losing mutants was at 45 h postinfection (hpi). In a reverse analysis, the likelihood of selecting a false mutant at these various time points was also obtained analyzing untreated wells (see Table S2 in the supplemental material). We found by using a *Z* score of 1.5, that 45 hpi similarly was the time point that showed the lowest likelihood of selecting a false mutant according to this less stringent criterion.

10.1128/mBio.02345-16.2 TABLE S1  Percentage of false negatives using *ufd1* siRNA. Download TABLE S1, DOCX file, 0.1 MB. Copyright © 2017 de Jesús et al. 2017 de Jesús et al. This content is distributed under the terms of the Creative Commons Attribution 4.0 International license.

10.1128/mBio.02345-16.3 TABLE S2  Percentage of wells showing a false positive. Download TABLE S2, DOCX file, 0.1 MB. Copyright © 2017 de Jesús et al. 2017 de Jesús et al. This content is distributed under the terms of the Creative Commons Attribution 4.0 International license.

### Identification of *D. melanogaster* host factors that modulate *Legionella pneumophila* intracellular replication.

More than 50% of the *D. melanogaster* annotated open reading frames (ORFs) (represented by ~12,144 dsRNAs from the *Drosophila* RNAi Screening Center, library 1.0 [28]) were analyzed, with duplicate assays performed in 384-well plates to identify host factors that either contribute to or interfere with intracellular replication (Fig. 1B) (29). Representations of both groups were selected as hits relative to the mean, and the *Z* scores were displayed (Fig. 1C). dsRNA target genes were considered for further analysis (hit) if (i) *L. pneumophila* intracellular replication was *Z* = 2 above the mean (increased replication hit) or *Z* = 1.5 below the mean (decreased replication hit) of the entire dsRNA-treated plate and (ii) the identical wells from both plates showed the same results. The data from the duplicate plates were then used to rank order the hits, and the gene annotations were acquired (30, 31) to determine the identity of the targeted genes. Afterward, the hits were subjected to a filtered examination, in which a subset of genes from individual functional groups was chosen for further analysis.

Among the 250 genes targeted in the secondary screen, genes encoding proteins involved in membrane trafficking, protein synthesis, cell cycle, and protein degradation were present in the collection. Each of these was then analyzed during intracellular growth at various times after bacterial challenge. The 250 cherry-picked dsRNAs were introduced into Kc167 cells for 4 days in duplicate plates, and cells were challenged with *L. pneumophila luxCDABE* (32, 33), with a plate containing untreated cells used as a control. dsRNAs that caused at least a 2-fold increase in the luciferase readout compared to untreated cells were selected for further study.

**FIG 2:**
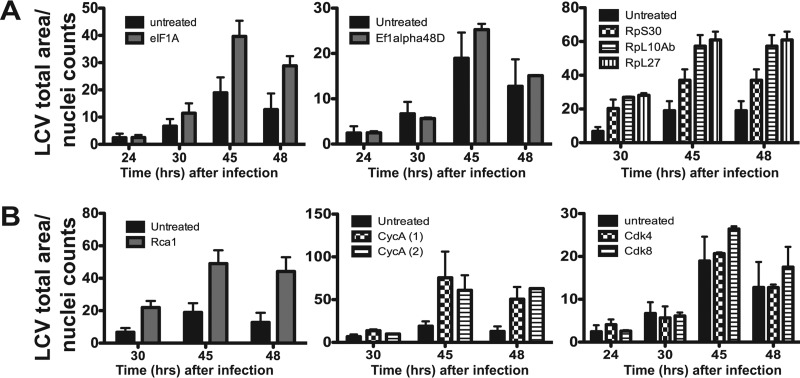
dsRNA depletion of transcripts encoding proteins involved in translation and the host cell cycle restrict *L. pneumophila* intracellular growth. Intracellular growth was determined by automated image analysis (Materials and Methods). *D. melanogaster* cells were treated with dsRNA directed against transcripts encoding translation factors (A) or cell cycle regulators (B) for 4 days and challenged with *L. pneumophila* GFP^+^ cells at an MOI of 1. Intracellular growth was measured at various times after challenge by calculating the total area of *Legionella* vacuoles relative to the area of cell nuclei. Numbers within parentheses denote the use of different dsRNAs for the same target gene.

Gene depletions that led to enhanced levels of *L. pneumophila* intracellular replication were of particular interest, because little is known about host restriction beyond the innate immune response. As predicted, depletion of innate immune factors, such as Imd, Myd88, Spz, and Dsor1, resulted in increased bacterial burden (Fig. 1D). Enhanced intracellular replication, however, was not limited to depletions predicted to disrupt the innate immune response. Strikingly, dsRNAs predicted to deplete proteins involved in cell cycle and host translation were also found to modulate the levels of *L. pneumophila* intracellular replication, although the ability to stimulate LCV growth was dependent on the particular gene targeted (Fig. 1D). Changing the assay from a luciferase readout to a ratio of total bacterial load relative to the number of host cell nuclei in each well yielded identical results (Fig. 2). Therefore, we decided to focus our analysis on these two networks.

### Host translation initiation and cell cycle progression modulate *Legionella pneumophila* intracellular replication.

Protein synthesis is coordinated by initiation, elongation, and termination of polypeptide chain synthesis by specific complexes. The initiation step begins with the assembly of the 43S initiation complex, comprised of GTP-bound eIF-2α (the α subunit of eukaryotic initiation factor 2), methionyl-tRNA, and the small ribosomal subunit (34, 35). A second complex then forms during cap-dependent translation as a consequence of eIF-4E, eIF-4A, eIF-4G, and the poly(A) binding protein (PABP) associating with the capped 5′ end of mRNA (34, 36). Immediately after these two complexes come in contact, they scan the mRNA for the start codon, leading to the hydrolysis of GTP bound to eIF-2α, formation of the 80S initiation complex, and subsequent peptide chain elongation (37).

**FIG 3:**
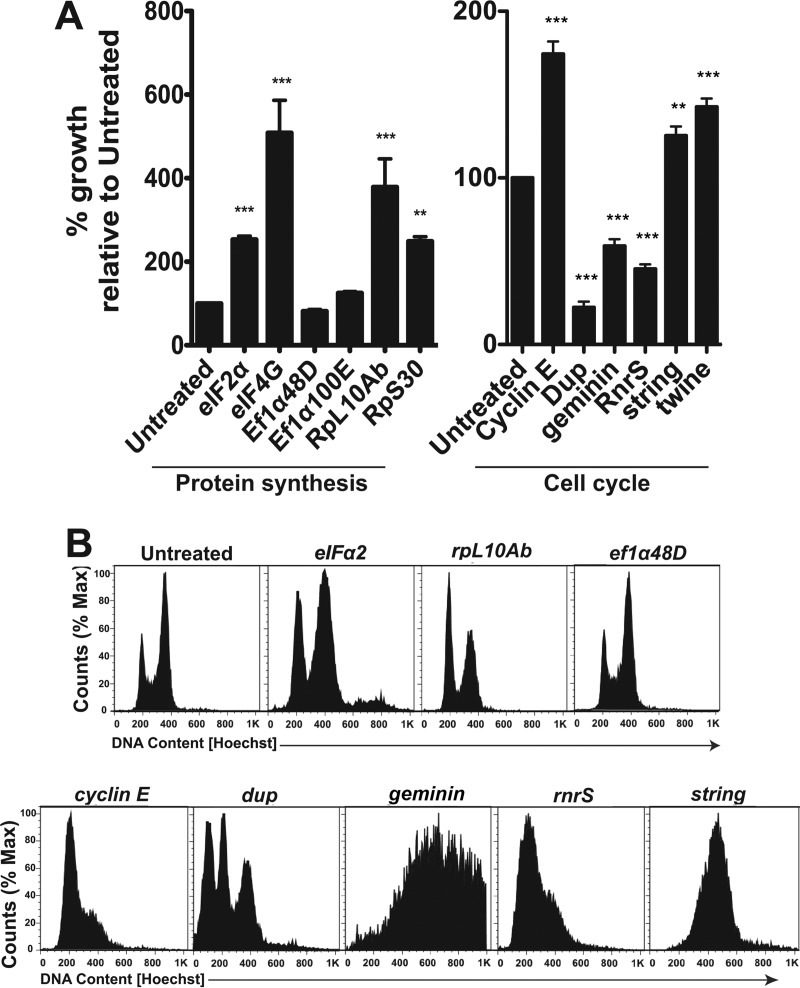
Cell cycle analysis of dsRNA-depleted Kc167 cells shows cell cycle disruption. (A) dsRNA-treated cells were challenged with Lp01 *lux*^*+*^ at an MOI of 1. Intracellular replication was measured 45 h after challenge. (B) *D. melanogaster* cells were treated with dsRNA against translation components (top panel) and the cell cycle control (bottom panel), followed by staining with 1 µg/ml Hoechst stain to determine DNA content using flow cytometry (Materials and Methods). **, *P* < 0.01, and ***, *P* < 0.001, compared to untreated cells by one-way analysis of variance (ANOVA) with *post hoc* Dunnett’s test.

Analyses of hits involved in protein translation revealed that *L. pneumophila* intracellular replication was enhanced in *D. melanogaster* cells depleted for translation initiation subunit genes (*eIF-2*α and *eIF-4G*) or when the small (*rpS30*) or large (*rpL10Ab*) ribosomal subunit genes were depleted (Fig. 2A and 3A). *L. pneumophila* prevents host protein synthesis by the concerted action of multiple T4SS-translocated proteins. At least three of these translocated proteins target the elongation factor eEF-1A (13, 17), which is involved in codon-anticodon base pairing during extension of the synthesized peptide (38). Depletion of either of the elongation factors Ef1α48D and Ef1α100E, the *D. melanogaster* homologues of human eEF-1A, does not have an effect on *L. pneumophila*’s ability to replicate inside the cells, indicating that stimulation of intracellular replication requires blockade at an early stage in protein synthesis (Fig. 3A).

The eukaryotic cell cycle is similarly highly regulated. It can be divided into four distinct phases. DNA is replicated during S phase, while in mitosis (M phase), chromosomes are distributed into new cells (39). The signals necessary to commit to S or M phase accumulate in G_1_ (prior to S phase) and G_2_ (prior to M phase) (39). The cell cycle responds to extracellular signals, and these stages are tightly regulated. Depending on the gene targeted, depletion of cell cycle regulators in *D. melanogaster* cells led to either increased or decreased *L. pneumophila* replication within the cells (Fig. 1D). For instance, depletion of genes involved in cell cycle progression at the G_2_/M phase (i.e., the Cdk8, Rca1, CycA, and string genes) resulted in enhanced levels of *L. pneumophila* replication, while depletion of the Cdk12 gene, which results in a blocked cell cycle progression in S phase, resulted in low levels of *L. pneumophila* replication compared to untreated cells.

To further determine if the cell cycle effects on *L. pneumophila* intracellular replication were dependent on the site of the dsRNA blockade, we depleted transcripts for well-characterized cell cycle components predicted to cause arrest at different stages of the cell cycle. Cyclin E (CycE) and its cognate cyclin-dependent kinase Cdk2 are required for the G_1_-S transition (40), while Cdt1 and geminin ensure that DNA replication occurs only once during S phase (41). Once in G_2_ phase, dephosphorylation of Cdks by Cdc25 phosphatases leads to mitosis (42). Arrest of *D. melanogaster* cells in G_1_ by depletion of CycE or in G_2_ by depletion of the Cdc25 phosphatase genes *string* and *twine* resulted in increased *L. pneumophila* yields (Fig. 3A). In contrast, arrest in S phase as a consequence of depletion of either *dup1* (the Cdt1 gene in mammalian cells), *geminin*, or *rnrS* resulted in depressed intracellular replication (43) (Fig. 2A and 3A).

As perturbations in the translation initiation machinery contribute to changes in cell cycle progression, we determined if dsRNA depletion of initiation factors resulted in cell cycle arrest (44–46). Untreated *Drosophila* cells showed the predicted accumulation of cells in G_2_, as observed previously using flow cytometric analyses (44). In contrast, depletion of *eIF-2*α and *rpL10Ab* increased the population of cells with DNA having G_1_ content, while depletion of the elongation factor gene *ef1α48D* yielded flow cytometric profiles indistinguishable from those of untreated controls (Fig. 3B). By way of comparison, depletion of *cycE* from cells resulted in the expected accumulation of cells with G_1_ content (Fig. 3B). Depletion of S phase regulators had a variety of cell cycle effects, ranging from loss of DNA content (*dup*), unregulated DNA synthesis (*geminin*), or accumulation at the S-M_1_ border (*rnrS*). Cells depleted of the G_2_ phosphatase gene *string* had increased G_2_ content within the cells (Fig. 3B), as previously reported (44). Taken together, these data are consistent with the model that arrest of the host cell cycle modulates *L. pneumophila* intracellular replication, with the consequences on bacterial yield dependent on the site at which the blockade occurs.

### Both G_1_ and G_2_/M are permissive for *L. pneumophila* targeting and replication.

**FIG 4:**
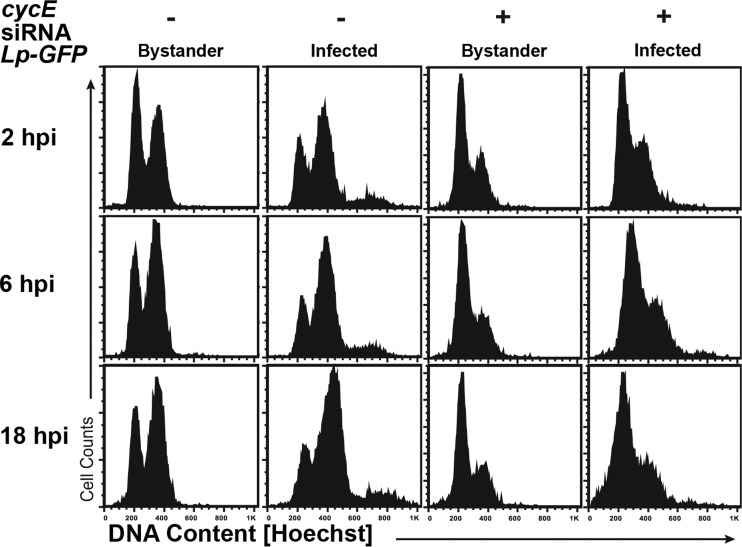
Bacterial targeting of either G_1_ or G_2_ allows *L. pneumophila* intracellular replication. *Drosophila* cells treated in the presence or absence of dsRNA directed against *cycE* were challenged with Lp01 GFP^+^, and DNA content was determined by Hoechst staining. Cells harboring bacteria (infected, GFP^+^) were gated from the uninfected population (bystander, GFP^−^) of the same sample based on GFP fluorescence. DNA levels within Kc167 cells were analyzed by flow cytometry.

To determine if *L. pneumophila* favors a particular phase for initial replication or infection, we analyzed *D. melanogaster* cell cycle dynamics after challenge with *L. pneumophila*. Proliferating Kc167 cells were incubated with *L. pneumophila* GFP^+^ cells, followed by DNA staining at various times after challenge (Materials and Methods). Cells associated with bacteria were separated from uninfected cells by flow cytometry based on GFP fluorescence, and DNA content was determined. Within the bystander-uninfected population (Fig. 4, *Lp-GFP* bystander), G_1_- and G_2_/M-phase cells were in roughly equal abundance, and this ratio did not vary from 2 to 18 h postinfection. Among cells harboring *L. pneumophila*, however, there was an enrichment of G_2_/M DNA, and as the infection progressed, there was increasing accumulation in this phase (Fig. 4, *Lp-GFP* Infected). The DNA content observed could be totally attributed to *Drosophila* DNA, because bacterial DNA did not contribute to the observed signal (see Fig. S1 in the supplemental material). The preferential infection of G_2_/M cells was not due to an inability to grow in G_1_ cells. To demonstrate this point, *D. melanogaster* cells were treated with dsRNA against *cycE*, predicted to block cells in G_1_. Depletion of CycE led to G_1_-phase arrest, and the cells harboring *L. pneumophila* largely had G_1_ DNA content throughout infection (Fig. 4, *cycE* dsRNA). Therefore, conditions that stimulate *L. pneumophila* replication, such as depletion of CycE, result in accumulation of bacteria in G_1_, indicating that both G_1_ and G_2_/M phases were hospitable targets for replication. Although initial infection of G_2_ cells was preferential, blocking cell cycle progression in either phase stimulated replication of the bacterium.

10.1128/mBio.02345-16.1 FIG S1  Cell cycle analyses of nuclei isolated from *Drosophila* cells challenged with *Legionella*/pGFP various times after uptake. Download FIG S1, PDF file, 0.2 MB. Copyright © 2017 de Jesús et al. 2017 de Jesús et al. This content is distributed under the terms of the Creative Commons Attribution 4.0 International license.

### S phase is restrictive for *L. pneumophila* intracellular replication.

**FIG 5:**
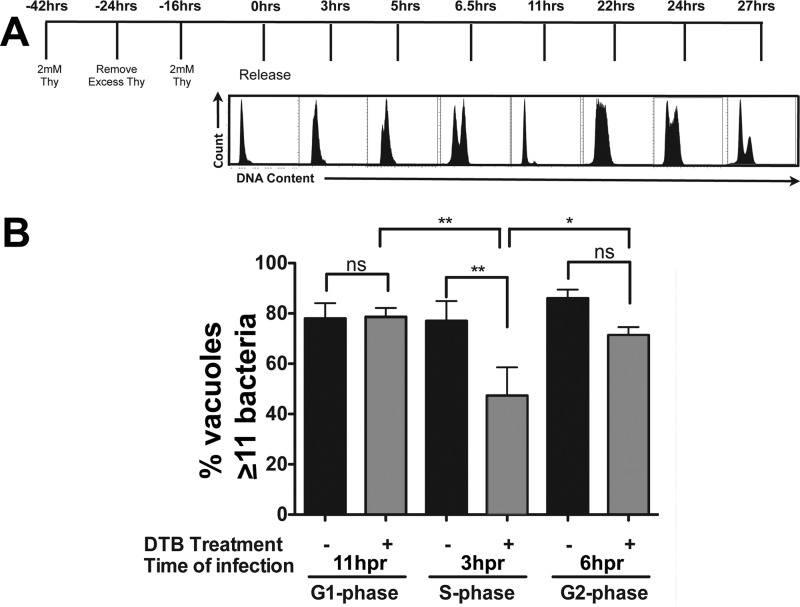
*Legionella pneumophila* intracellular replication is diminished in S phase HeLa cells. (A) HeLa cells were synchronized by a double-thymidine block (DTB [−42 to 0 h]), released, and challenged with *L. pneumophila* at 3, 6, and 11 h postrelease (Materials and Methods). Histograms represent the cell cycle profile of Hoechst-stained cells at the indicated times postrelease (hpr). (B) *L. pneumophila* cells show defective growth in S phase cells. Lp01 was used to challenge HeLa cells at the noted time points, and the noted cell cycle phases were determined based on flow analysis. Cells were fixed and permeabilized 14.5 h after challenge and stained with anti-*Legionella*, and the numbers of bacteria per vacuole were scored microscopically, displaying the number of vacuoles having more than 11 bacteria. ns, not significant; *, *P* ≤ 0.05, and **, *P* < 0.01, by one-way ANOVA with *post hoc* Bonferroni’s multiple-comparison test.

To determine if intracellular growth of *L. pneumophila* in mammalian cells is also controlled by cell cycle dynamics, HeLa cells were challenged with bacteria at each phase of the cell cycle (Fig. 5). To this end, HeLa cells were synchronized using the double-thymidine block strategy over a 42-h period, and bacteria were added at various times after release to allow infection at specific stages in the cell cycle (47). At 14.5 h postinfection (hpi), the cells were then fixed and LCV formation was determined by microscopy (Materials and Methods). HeLa cells that were challenged with *L. pneumophila* 3 h postrelease had a large population of cells in S phase (Fig. 5). There was a clear decrease in the ability to support bacterial replication relative to cells that had not been synchronized (Fig. 5, S phase). In contrast, cells challenged 6 h postrelease (when there was a distribution of HeLa cells in all three phases) or 11 h postrelease (when the cells were mainly in G_1_) showed no such defect (Fig. 5). These data support the model from dsRNA depletion of *Drosophila* cells that S phase cells are either inefficient at supporting *L. pneumophila* replication or may directly interfere with formation of replication compartments. Therefore, while uptake of bacteria in the G_1_ and G_2_/M phases appears permissive for *L. pneumophila* replication, S phase appears particularly restrictive, with bacteria internalized in S phase showing reduced replication competence relative to other phases.

### The integrity of the LCV is compromised in host cells targeted in S phase.

During S phase, the nuclear envelope expands to accommodate the newly synthesized DNA (48–50). Of potential importance to *L. pneumophila* replication is the fact that the nuclear envelope is connected to the endoplasmic reticulum (ER) (49). In view of the association of the *L. pneumophila*-containing vacuole with the ER, we hypothesized that nuclear envelope expansion affects the integrity of the *L. pneumophila*-containing vacuole, interfering with intracellular replication. Previous work had shown that bacterial strains harboring lesions in either *sdhA* or the *lidA wipB* double mutant were unable to maintain LCV integrity after uptake into cells, resulting in defective intracellular growth. This argues that loss of LCV integrity is a potential Achilles heel for establishing an intracellular replication site (14, 15).

**FIG 6:**
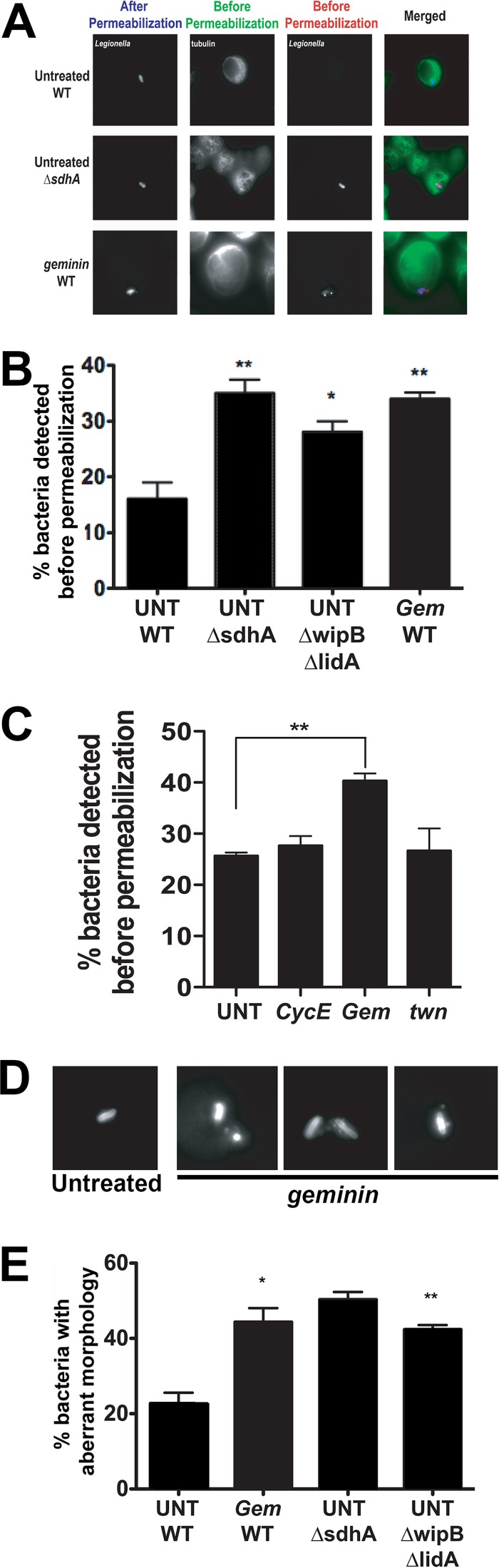
S phase arrest by *geminin* leads to instability of the *Legionella* vacuole. *D. melanogaster* cells were treated with dsRNA against *geminin* and challenged with *L. pneumophila* for 18.5 h (A) Cells were stained with anti-*Legionella* before (red) and after (blue) permeabilization. α-Tubulin (green) was stained as a cytoplasmic marker. (B) Comparison of *geminin* (*Gem*) dsRNA treatment to bacterial mutations known to result in enhanced bacterial permeability. Displayed are the percentages of total associated bacteria that can be detected before chemical permeabilization. UNT, untreated. (C) Depressed vacuole integrity is specific to arrest in S phase. Displayed are cells arrested in the G_1_ (*cycE*), S (*Gem*), and G_2_/M (*Twn* [*twine*]) phases. (D) Example of *L. pneumophila* cells within untreated or *geminin*-depleted cells. (E) Quantification of the number of bacteria with aberrant morphology, based on images displayed in panel D. *, *P* < 0.05, and **, *P* < 0.01, compared to untreated cells infected with wild-type bacteria by one-way ANOVA with *post hoc* Dunnett’s test.

To determine if the integrity of the LCV is compromised during infections of host cells present in S phase, the ability of an antibody directed against *Legionella* to penetrate the vacuole was determined microscopically after fixation in either the absence or presence of chemical permeabilization. The LCV established by wild-type (WT) *L. pneumophila* maintains a barrier against antibody probing in the presence of fixation (14, 15), in contrast to the plasma membrane, which allows cytoskeletal components such as α-tubulin to be detected after fixation in the absence of permeabilization (Fig. 6A, Before Permeabilization). In contrast to controls, vacuoles established in *D. melanogaster* cells depleted of *geminin* were permeable to antibody probing, with levels of antibody accessibility to the bacteria similar to that observed for untreated cells harboring either the Δ*sdhA* or Δ*wipB* Δ*lidA* mutant (Fig. 6A and B). Vacuole permeability was specific for cells that were arrested in S phase, as depletion of *cycE* or *twine*, G_1_- and G_2_-phase cell cycle regulator genes, respectively, did not cause an increase in the number of bacteria that could be detected before permeabilization (Fig. 6C). These results indicate that the integrity of the *L. pneumophila*-containing vacuole is reduced in host cells present in S phase, but not when the host cells are in the G_1_ or G_2_/M phase.

Cytosolic exposure of a Δ*sdhA* mutant as a consequence of vacuole disruption can lead to leakage of bacterial component into the host cytosol, activation of cytoplasmic innate immune responses, and bacterial degradation (6, 16). Therefore, we examined the levels of bacterial degradation within these cells. During infections in *geminin*-depleted cells, *L. pneumophila* had a variety of morphological characteristics (such as punctate staining) that were quite distinct from the rod shapes seen during infections in untreated cells (Fig. 6D) (14). Upon quantification, we found that a high percentage of the *L. pneumophila* cells contained within disrupted vacuoles in *geminin*-depleted cells had forms consistent with degradation compared to bacteria in untreated cells (Fig. 6E). Consequently, there is a direct connection between replication defects observed during S phase arrest and the presence of an unstable LCV that results in exposure to the host cytosol and bacterial degradation.

### *L. pneumophila* challenge of mammalian cells in S phase results in Icm/Dot-dependent cell cycle arrest and LCV degradation.

**FIG 7:**
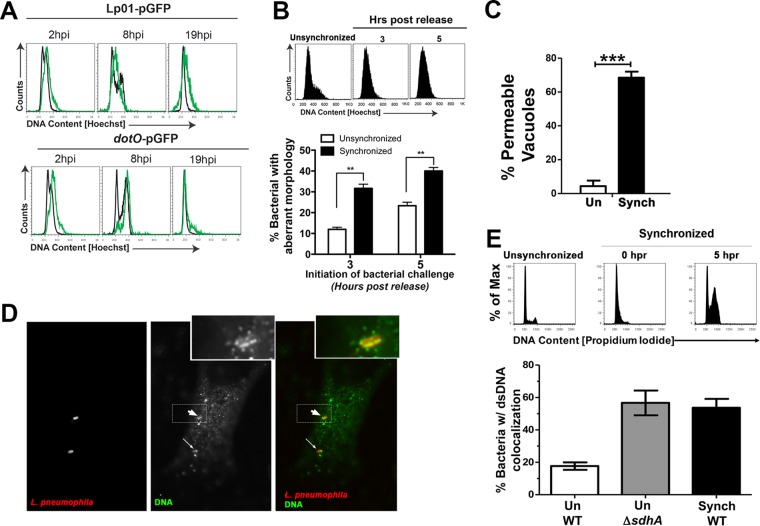
*L. pneumophila* cell cycle arrest in S phase results in loss of vacuole integrity. (A) HeLa cells were synchronized by the double-thymidine block method and challenged with wild-type *L. pneumophila*/pGFP (Lp01) or the Δ*dotO*/pGFP mutant 3 h after release. Various times after uptake, cells were collected and analyzed by flow cytometry to determine the cell cycle profile of Hoechst-stained cells. Infected cells were separated from within the total population based on GFP fluorescence. Black lines indicate uninfected cells and green lines infected cells. (B) Challenge of S phase cells with *L. pneumophila* results in bacterial degradation. HeLa cells were synchronized by double-thymidine block and challenged with *L. pneumophila* Lp01 3 or 5 h after release. (Top panel) Hoechst staining of cells in the absence of synchronization or at noted times of release. The infection was then allowed to proceed for 6 h, followed by fixation and staining with anti-*L. pneumophila*. (Bottom panel) The number of bacteria with aberrant morphology was scored visually as in Fig. 6D (Materials and Methods). (C) Challenge of S phase cells results in permeable LCVs. HeLa cells were synchronized as in panel B, and the cells were released for 3 h prior to challenge with Lp01. At 6 h postinfection, the cells were fixed, and vacuole integrity was determined by probing with anti-*L. pneumophila* in the absence of chemical permeabilization (Materials and Methods). (D) Detection of bacterium-associated DNA in S phase cells. Synchronization was performed as in panel B, and cells were challenged with Lp01 5 h postrelease. Six hours later, the samples were fixed, probed with anti-DNA (green), permeabilized, and then probed with anti-*L. pneumophila* (red) and analyzed by immunofluorescence microscopy. The dashed box represents area that is magnified in the inset, with the fat arrow pointing to an internalized bacterium. Insets were artificially magnified by a 4.167-fold increase in the pixel density. The thin arrow points to a second internalized bacterium. (E) Challenge of S phase cells with *L. pneumophila* results in exposure of bacterium-associated DNA. Cells synchronized by double-thymidine block were released for 5 h (top panel), challenged for 6 h, and probed as in panel D, and the fraction of bacteria showing DNA association was determined by immunofluorescence microscopy (bottom panel). Un WT, unsynchronized cells challenged with WT; Un ΔsdhA, unsynchronized cells challenged with the Δ*sdhA* mutant; Synch WT, synchronized cells challenged 5 h after release with the WT.

Cell cycle arrest and consequent bacterial degradation could explain why synchronized cells challenged in S phase are poor targets for intracellular replication. Therefore, HeLa cells were synchronized by double-thymidine block and challenged with bacteria at appropriate times after release to determine if *L. pneumophila* blocks the host cell cycle (47). Synchronized HeLa cells were challenged with *L. pneumophila* GFP^+^ strains at time points corresponding to early S phase, and the DNA content within the cells harboring *L. pneumophila* was compared by flow cytometry to that of bystander-uninfected cells (Fig. 7A). Bystander, uninfected HeLa cells were able to progress through the cell cycle from 2 h to 18 h postinfection (Fig. 7A, black lines). In contrast, cells harboring WT *L. pneumophila* GFP^+^ cells appeared locked in S phase, and there was little progression from 2 to 19 hpi (Fig. 7A, green lines). HeLa cells harboring the type IV secretion system-deficient *dotO* mutant progressed from S phase through G_2_ and back to G_1_ phase (Fig. 7A, green lines, *dotO-*pGFP). Therefore, S phase cells challenged with *L. pneumophila* were arrested in a fashion dependent on the Dot/Icm secretion system.

We next determined if Icm/Dot-dependent S phase arrest resulted in loss of LCV integrity and subsequent bacterial degradation. HeLa cells were synchronized, and at 3 and 5 h after release, cells were challenged with *L. pneumophila* to determine the levels of bacterial degradation at 6 hpi. By 3 h postrelease, there was a large fraction of cells in S phase (Fig. 7B). When HeLa cells were challenged at either of these two time points, there was an increase in bacteria with aberrant morphology compared to unsynchronized controls, particularly when challenged at 3 h postrelease (Fig. 7B). Based on the 3-h release being an effective strategy to identify S phase, we analyzed cells after 3 h postrelease for membrane integrity (Fig. 7C). The vast majority of cells synchronized in this fashion harbored LCVs that had lost membrane integrity, as determined by identifying permeable vacuoles and quantitating them using the antibody accessibility assay. To determine if the aberrant morphology of bacteria was connected to loss of bacterial integrity and liberation of microbial DNA, samples were probed with anti-DNA followed by observation with immunofluorescence microscopy (Fig. 7D). There was considerable punctate staining throughout the cytoplasm in all cells, presumably due to mitochondrial DNA, so staining was scored by identifying bacteria with anti-*L. pneumophila* and scoring whether the anti-DNA probe revealed antigen that encompassed or appeared to be extruded from the bacteria (Fig. 7D, insets). Using this approach in unsynchronized cells, there was a 3-fold increase in bacterium-associated DNA staining in cells challenged with the Δ*sdhA* mutant compared to the wild-type control, consistent with previous results based on morphological scoring (Fig. 7E) (14). Also consistent with the morphological assay, over 50% of the cell-associated bacteria showed evidence of liberated DNA after a 6-h incubation with synchronized S phase cells (Fig. 7E). These results indicate that *L. pneumophila* interference of progression through S phase results in loss of LCV integrity and degradation of exposed bacteria.

## DISCUSSION

*L. pneumophila* exploits multiple pathways within hosts in order to replicate intracellularly, although there has been little effort to identify factors outside innate immunity that could negatively modulate intracellular growth. We took the approach of identifying these factors by screening for dsRNA that enhanced replication of the bacterium in *Drosophila* cells. Unexpectedly, interference of translation initiation stimulated intracellular replication, as did disruption of the cell cycle at specific sites. Specifically, growth arrest in G_1_ or G_2_/M enhanced *L. pneumophila* replication, whereas arrest in S phase or introduction of bacteria onto synchronized S phase cells depressed growth. These observations have special significance regarding the ecology of *L. pneumophila*. The infectious reservoir consists of amoebae harboring the microorganism within aquatic environments prior to replication of bacteria in alveolar macrophages (51). We hypothesize that within the natural aquatic habitat, amoebae are likely to be growing slowly, or perhaps even growth arrested, due to depressed nutrients. As a consequence, S phase would be initiated infrequently, providing conditions that are ideal for replication of *L. pneumophila*. We have observed previously that nutrient deprivation of dividing host cells can stimulate intracellular replication, as maximal replication of *L. pneumophila* within the model amoebal species *Dictyostelium discoideum* is stimulated in glucose-free medium. These conditions reduce the rate of mitosis of *D. discoideum* without causing a developmental switch that leads to sporulation (52).

In this study, we found that *L. pneumophila* replication proficiencies within *Drosophila* cells in either G_1_ or G_2_/M phases were indistinguishable and that locking the cell in either of these cell cycle stages using siRNA depletion stimulated intracellular growth. *L. pneumophila* is able to encounter a number of different hosts in the environment, which may have very different strategies for cell cycle control or arrest under nutrient-limiting conditions, so it is possible that *L. pneumophila* has evolved strategies that would allow it to proliferate within hosts that are in either G_1_ or G_2_/M stages at the time of bacterial infection. Due to the fact that amoebal species are often found in environments in which there is little division, we hypothesize that *L. pneumophila* has developed strategies to allow growth in nondividing amoebae, serving the pathogen well during encounters with human macrophages. This is clearly an advantage for a bacterium that infects nondividing immune cells, but it could also be a strategy that allows hosts to interfere with intracellular replication of a pathogen. Macrophages may have an unappreciated strategy of limiting pathogen replication, as under the appropriate stimuli, macrophages have the capacity to initiate a replication cycle and, in turn, disrupt intracellular growth (53, 54).

The eukaryotic cell cycle is a known target of viral and bacterial pathogens, which either stimulate cell division or prevent it (55, 56). Evidence is presented here that after *L. pneumophila* enters into S phase cells, the host cell cycle is arrested. These results are consistent with recent observations in *Acanthamoeba castellanii*, which were shown to be blocked from synthesizing DNA after *L. pneumophila* challenge, consistent with a cell cycle arrest (27). Bacterial pathogens encoding type III secretion systems such as *Escherichia coli*, *Shigella*, and *Burkholderia* translocate protein toxins into the host in order to modulate their host cell cycle (57–59). Although no homologues of these toxins are found in *L. pneumophila*, cell cycle arrest is probably not limited to S phase. It is already well established that cells infected with virulent *L. pneumophila* have depressed protein synthesis, which results from either Icm/Dot-translocated translation elongation inhibitors or a cell-intrinsic pathogen response that blocks translation initiation (13, 17, 18, 20, 60). Inhibition of translation at either of these steps is likely to provide the necessary machinery to cause arrest at multiple points in the cell cycle. As shown here, depletion of host cell translation initiation complexes causes cell cycle arrest in G_1_, consistent with results from a previous study (44). Furthermore, it has long been established that chemical inhibition of protein elongation blocks mitosis, preventing exit from G_2_ (61). Thus, these observations are compatible with the idea that *Legionella* proteins responsible for host protein synthesis inhibition could constitute a mechanism that the bacterium employs in order to manipulate the host cell cycle and stimulate growth in dividing cells (18). The idea that bacterial translation inhibitors are important growth promoters would be similar to the strategy observed during *Pseudomonas entomophila* infections of *Drosophila*, in which inhibition of translation initiation within gut cells prevents epithelial cell renewal and supports bacterial growth (62). How this occurs remains unresolved.

The results here provide an explanation for why intracellular growth is depressed in S phase. *L. pneumophila*-containing vacuoles (LCVs) established during S phase showed increased permeability. Furthermore, defective vacuoles in S phase were associated with the appearance of bacterial cells having altered morphologies, indicating cytoplasmic degradation of the microorganism. These phenotypes were reminiscent of vacuoles harboring *L. pneumophila* Δ*sdhA* mutants (14). SdhA is a bacterial protein that helps maintain the integrity of the replication compartment, possibly by recruiting membranes or modifying the lipid composition of the LCV. It is possible that S phase-specific membrane trafficking could disrupt the LCV lipid profile, or the demands of S phase on lipid pools could result in lipid starvation of the LCV. The defective vacuoles observed in these cells are particularly striking because the bacterium is bringing a full load of effectors, indicating that S phase progression could be a strategy that allows host restriction of intravacuolar pathogens.

In summary, these results indicate that an important host cell-intrinsic strategy to interfere with intracellular growth is proliferation of the cell cycle through S phase and possibly through mitosis. Although we have blocked proliferation through the introduction of dsRNAs that directly interfere with either the cell cycle or translation initiation, arrest is presumably induced in the environment by nutrient starvation conditions or by targeting of terminally differentiated cells such as macrophages, setting up host cells that are perfectly situated to support intracellular replication. Surprisingly, introduction of bacteria into S phase cells resulted in instability of the replication vacuole, a phenomenon that had only been observed with bacterial mutants defective for a specific class of translocated proteins (14). The fact that dsRNA causing arrest in either G_1_ or G_2_/M stimulated intracellular growth in proliferating host cells argues that the organism shows no preference for either phase. Rather, the effects could be explained by preventing host cells from entering S phase, which would interfere with intracellular growth and destabilize the LCV. Future work will be devoted to understanding why the *L. pneumophila* replication site is sensitive to events during cell cycle progression and, in particular, the events that cause degradation of the LCV.

## MATERIALS AND METHODS

### Bacterial strains, plasmids, and media.

All strains and plasmids used in this study are described in Table S3 in the supplemental material. The *Legionella pneumophila* strains used in this study were derived from the Philadelphia 1 isolate. Strain Lp01 is streptomycin resistant and intracellular growth competent (63, 64). Lp02 is a thymidine autoxotroph that is streptomycin resistant and intracellular growth competent (63, 64). Lp03 is a derivative of Lp02 with the *dotA3* point mutation and is defective for intracellular replication (63). The *dotO* strain is a derivative of Lp01 and is defective for intracellular replication (65). For the Lp02 and Lp03 strains, the chromosomal *thyA* mutant allele was replaced with the *thyA*^+^ allele by allelic exchange (66). The plasmid pAM239 encodes chloramphenicol resistance and allows production of the green fluorescent protein (GFP) from the P_*tac*_ promoter, which is inducible by isopropyl-β-d-thiogalactopyranoside (IPTG) (52, 67). *L. pneumophila* was grown on plates containing charcoal and yeast extract buffered with ACES [*N*-(2-acetamido)-2-aminoethanesulfonic acid; Sigma] adjusted to pH 6.9 and supplemented with 0.4 mg/ml of l-cysteine, and 0.135 mg/ml of ferric nitrate (CYE), as well as 0.1 mg/ml thymidine when necessary (Sigma) (68). Liquid cultures of *L. pneumophila* were prepared in the same medium, but without charcoal and agar (AYE). Overnight cultures of *Legionella* were prepared by serially diluting cultures 1:2 in AYE supplemented with the appropriate antibiotic and incubated at 37°C with shaking. Chloramphenicol was used at 5 µg/ml. For infections, overnight cultures were used and all strains were grown to the postexponential phase (*A*_600_ of 3.5 to 4.0). The approximate concentration of bacteria was determined by assuming that an *A*_600_ of 1.0 is equivalent to 10^9^ bacteria/ml.

10.1128/mBio.02345-16.4 TABLE S3  Strains, plasmids, and oligonucleotides. Download TABLE S3, DOCX file, 0.1 MB. Copyright © 2017 de Jesús et al. 2017 de Jesús et al. This content is distributed under the terms of the Creative Commons Attribution 4.0 International license.

### Cell culture.

Routine propagation of cultured *Drosophila melanogaster* Kc167 cells was performed as described previously (26, 68) using 1× Schneider’s *Drosophila* medium (Thermo Fisher Scientific), supplemented with 10% heat-inactivated fetal bovine serum (HI-FBS; Gibco) at ~24°C. HeLa cells were cultured at 37°C with 5% CO_2_ and routinely grown in Dulbecco’s modified Eagle’s medium (DMEM) with a high glucose concentration, l-glutamine, phenol red, and sodium pyruvate, supplemented with 10% HI-FBS (Gibco).

### Infections using *Legionella pneumophila.*

Challenge of host cells was carried out with motile bacteria at the appropriate multiplicity of infection (MOI), depending on the specific experimental protocol (see Table S4 in the supplemental material). After challenge, the infection plates were placed in the centrifuge for 5 min at 200 × *g* to synchronize the infection. Cell monolayers were washed twice with the appropriate media and supplements either 1 h (Kc167) or 2 h (HeLa) after challenge.

10.1128/mBio.02345-16.5 TABLE S4  Multiplicity of infection (MOI) used per assay. Download TABLE S4, DOCX file, 0.1 MB. Copyright © 2017 de Jesús et al. 2017 de Jesús et al. This content is distributed under the terms of the Creative Commons Attribution 4.0 International license.

### Vacuole size measurements.

Kc167 cells were challenged with *L. pneumophila* GFP^+^ cells and treated with 10 µg/ml of erythromycin at 19 h after challenge. Infections were allowed to proceed for an additional 11 or 21 h, at which point, the cells were fixed in 3.7% paraformaldehyde in 1× PBS and stained with 1 µg/ml Hoechst stain. Images were taken using a Molecular Devices ImageXpress automated microscope with a Nikon 20× Plan Apo lens for 2 sites per well using the DAPI (4′,6-diamidino-2-phenylindole) and FITC (fluorescein isothiocyanate) optimized filter sets. Downloaded images were subjected to an automated MetaXpress script that counts for the total area of cell nuclei (based on Hoechst staining) and bacterial replication (based on total GFP signal).

### Primary dsRNA screen.

A dsRNA pool, constructed by the *Drosophila* RNAi Screening Center (Harvard Medical School), was used to perform high-throughput screening in *D. melanogaster* cells (30, 31). Briefly, Kc167 cells were resuspended in Schneider’s *Drosophila* medium without FBS, and 2.5 × 10^4^ cells in 25 µl were plated in each well of 384-well plates containing dsRNA. The plates were placed in the centrifuge for 1 min at 200 × *g*, followed by incubation at ~24.5°C in a humid chamber. After 45 min, 1 volume of Schneider’s medium supplemented with 10% HI-FBS was added to each well. Cells were incubated for 4 days before infection. Treatment was performed in duplicate. dsRNA-treated cells were then challenged with Lp01/pAM239(GFP^+^) in the presence of chloramphenicol (5µg/ml) and IPTG (0.1 mM). Infection was allowed to proceed for 45 h. The wells were then fixed in a high-throughput format with 3.7% formaldehyde in 1× PBS for 30 min at room temperature, followed by washing with PBS. Cell nuclei were stained with 1 µg/ml of Hoechst stain-PBS for 10 min at room temperature. Plates were stored at 4°C until analysis.

### Data acquisition and analysis.

Images of each well were captured using ImageXpress (Molecular Devices) (69). All images were captured using 20× Plan Apo lens, with 2 fields per well, using the DAPI and FITC channels. The “Count nuclei” function from the MetaXpress software was used to calculate the pixel area of the total cell number and the *Legionella*-containing vacuole (LCV) number. In images from the DAPI channel, a nucleus was considered positive if it had a minimum width of 1 µm and a maximum width of 12.5 µm with a pixel intensity of 600 gray levels above background. LCVs were counted as positive if, after 45 h of growth, they were larger than 1 µm and had a pixel intensity of 1,600 gray levels above background. The number of particles per image and their pixel areas were recorded and analyzed using Microsoft Excel.

The mean LCV pixel area and the mean ratio of LCV area over total number of cell nuclei were calculated for each plate and for individual wells containing dsRNAs. Infections on cells with dsRNAs whose total LCV area was above 2 or below 1.5 standard deviations (SD) from the mean pixel area of the entire plate treated with dsRNA were selected as “positive.” In addition, dsRNAs that were 2 SD below in just one replicate plate were selected as “mild-effect” hits (*Z* score of >2). For instance, if depletion of a dsRNA-targeted gene led to levels of replication showing a *Z* score of >2 in one plate, but it showed a less severe defect in the second plate, the mean ratio of the dsRNAs between both plates was calculated. If the mean ratio of the corresponding wells showed a *Z* score of >1, then the dsRNA target gene was selected as a mild effect hit and studied further in secondary screens.

In order to rank order the hits, we determined the bacterial replication efficiency relative to the mean of the plate. The ratio of total LCV pixel area to number of cell nuclei for each dsRNA relative to the mean of the plate was calculated for each well. The average ratio of the duplicate plates was obtained and used to rank order the hits.

### Filtering of hits of interest.

dsRNAs were eliminated from further evaluation if they were represented in any of the following categories: (i) the dsRNA had a predicted off-target (based on having ≥10 bp matches [70]), (ii) the dsRNA represented internal controls; (iii) the dsRNA targeted a *Drosophila* gene that had not been annotated; (iv) the dsRNA concentration was low; or (v) the dsRNA-targeted gene did not have a predicted protein domain, human homologue, or *Drosophila* gene name.

### Secondary screen.

*L. pneumophila lux*^*+*^ cells harboring the *Photorhabdus luminescens luxCDABE* operon under the control of the *ahpC* promoter (32), was grown overnight in AYE medium supplemented with kanamycin (20 µg/ml). Infections were carried out at a multiplicity of infection (MOI) of 1. After challenge, cells were placed in the centrifuge for 5 min at 200 × *g* to synchronize the infection prior to incubation at the appropriate temperature. After 1 h of incubation, cells were washed twice with Schneider’s medium supplemented with 10% HI-FBS. Infection was allowed to proceed for up to 48 h. At each time point, luminescence was recorded using a SpectraMax M5 plate reader with an integration time of 1,500 ms. The *Z* score for each dsRNA was determined relative to the mean relative luminescent units (RLU) obtained from infections in an untreated control plate at the same indicated time points.

### dsRNA construction.

dsRNAs were constructed as previously described (68, 71). Briefly, genes of interest were amplified with *Drosophila* genomic DNA by PCR using standard PCR reagents and conditions and the appropriate oligonucleotides (Table S3). Amplified fragments were purified using a PCR purification kit (Qiagen) following the manufacturer’s protocol. To synthesize the dsRNA, the purified amplicon was used as a template using the MEGAscript RNAi kit from Ambion following the manufacturer’s protocol. Newly synthetized dsRNA was stored at −20°C until used. To test target gene depletion after dsRNA treatment, quantitative reverse transcription-PCR (qRT-PCR) was carried out as previously described (68).

### Flow cytometry analyses.

A total of 1 × 10^6^ *D. melanogaster* Kc167 cells or 2.5 × 10^5^ HeLa cells were seeded per well of a 12- or 6-well plate, respectively. Cells were challenged at the specified multiplicities of infection (MOI) described in Table S4. At the appropriate time after challenge, cells were washed twice with 1× PBS, incubated with 0.05% trypsin-EDTA for 1 min, and lifted with the appropriate medium supplemented with 10% HI-FBS. Cells were collected by centrifugation for 5 min at 200 × *g* at room temperature. Kc167 cells were resuspended in 1 µg/ml Hoechst stain in 1× PBS and incubated at room temperature for 45 min, while HeLa cells were resuspended in 5 µg/µl Hoechst stain in 1× PBS and incubated at 37°C for 30 min. For propidium iodide (PI) staining, samples were incubated in 100% ethanol at 4°C, pelleted for 5 min at 1000 rpm in a microcentrifuge, washed in PBS, and incubated for 30 min at 37°C in PI cocktail (30 g/ml PI, 1:100 RNase [Thermo-Fischer Scientific AM2286] in PBS). Cells were pelleted, resuspended in PI cocktail, and subjected to flow cytometry. Flow cytometry analyses were carried using a BD LSR II flow cytometer. Data were analyzed using FlowJo version 6.4.7 or 7.6.5.

### HeLa cell cycle synchronization.

To perform double-thymidine addition blocks, HeLa cells were synchronized by incubating 1 × 10^6^ cells in a 10-cm^2^ dish with an excess of 2 mM thymidine for 18 h. Cells were washed two times with 1× PBS and incubated in DMEM-FBS without thymidine for 8 h, followed by a second exposure to 2 mM thymidine (47). After 14 to 16 h, cells were collected and replated at 2.5 × 10^5^ cells/well in 6-well plates for flow cytometry or 1 × 10^5^ cells/well in a 24-well plate containing coverslips for immunofluorescence studies. Cells were infected at the indicated times after release.

### Immunofluorescence analysis of *L. pneumophila*-infected cells.

A total of 1 × 10^6^ Kc167 cells/well were plated overnight in a 24-well plate containing concanavalin A-coated coverslips in 1× Schneider’s *Drosophila* medium supplemented with 10% FBS prior to challenge with Lp01 (72). For HeLa cell infections, 2 × 10^5^ HeLa cells/well were plated in 24-well plates containing coverslips in DMEM supplemented with 10% HI-FBS, and cells were challenged by bacteria either after overnight incubation or at the appropriate time after synchronization release (Table S4).

At the appropriate times after challenge, cells were washed with PBS, fixed in 4% paraformaldehyde in 1 × PBS for 30 min at room temperature for *Drosophila* cells or at 37°C for HeLa cells, and permeabilized with −20°C methanol. Nonspecific binding was blocked with 4% bovine serum albumin (BSA) in 1× PBS at room temperature, and intracellular *Legionella* was stained with rabbit anti-*L. pneumophila* Philadelphia 1, followed by detection with goat anti-rabbit IgG conjugated with Alexa Fluor 488 or Alexa Fluor 594 (Molecular Probes). The number of bacteria within vacuoles was scored visually for at least 100 vacuoles per coverslip using 100× objectives.

Vacuole instability was assayed as previously described (14). Briefly, cells challenged with bacteria were fixed as described above. Cytosolically exposed bacteria and intracellular markers were stained before permeabilization using anti-*L. pneumophila* and anti-α-tubulin (Sigma) followed by detection with goat anti-rabbit IgG-Alexa Fluor 350 and goat anti-mouse IgG-Alexa Fluor 488 (Molecular Probes), respectively. Cells were permeabilized with cold methanol for 20 min, and intracellular bacteria were stained with anti-*Legionella* antibody followed by goat anti-rabbit IgG-Alexa Fluor 594 (Molecular Probes). Bacteria with aberrant morphologies were evaluated in cells challenged with bacteria at various times in the host cell cycle. Aberrant bacteria were defined as being rounded, rough, or with punctate or blebbing morphologies, as opposed to rod shaped (14, 15). At least 100 vacuoles were scored per coverslip.

For detection of DNA, fixed samples were probed by incubation for 1 h at 37°C with rabbit anti-*L. pneumophila* (1:20,000) and mouse anti-dsDNA (ab27156, 1:1,000; Abcam, Inc.) in PBS plus 4% BSA. The samples were washed 3× with 4% BSA–PBS and incubated for 1 h at 37C with goat anti-rabbit-Alexa Fluor 594 (1:500; Molecular Probes) and donkey anti-mouse-Alexa Fluor 488 (1:500; Molecular Probes). Samples were then washed and permeabilized with ice-cold methanol for 20 s prior to being probed with anti-*L. pneumophila* and detection with goat anti-rabbit-Alexa Fluor 350 (1:500; Molecular Probes).
