# Supplementary material for: Host Cell S Phase Restricts Legionella pneumophila Intracellular Replication by Destabilizing the Membrane-Bound Replication Compartment
Source: mBio. 2017 Aug 22;8(4):e02345-16. doi: 10.1128/mBio.02345-16 (PMC5565972; doi:10.1128/mBio.02345-16)
Supplement: TABLE S1 [file mbo004173448st1.docx]

**Table S1. Percentage of false negatives using *ufd1* siRNA.**

| ***z* score** | **40 hrs** | **45 hrs** | **48 hrs** |
| --- | --- | --- | --- |
| **1** | 33% | 0% | 29% |
| **1.5** | 57% | **9%** | 90% |
| **2** | 95% | 64% | 100% |

Strain Lp01 was used to challenge Drosophila Kc1167 cells seeded in 300 wells for the times noted (35-48 hrs). The ratio of areas of LCV/cell nuclei was determined for the 300 wells, and the *z* score was determined from the standard deviation (sd). In parallel, 23 wells of Kc1167 cells were treated with siRNA directed against *ufd1*, which is known to cause defective intracellular growth ([1](#_ENREF_1)). The data are expressed as the percentage of *ufd1* siRNA-treated wells that were false negatives, given the value of the *z* score determined from the untreated wells. For instance, at 45 hpi, treatment with *ufd1* dsRNA revealed that 9% of wells would be lost if a cutoff were used that required the

siRNA to have an effect on intracellular growth greater than 1.5 x sd from the mean.

**Reference**:

1. **Dorer MS, Kirton D, Bader JS, Isberg RR.** 2006. RNA interference analysis of Legionella in Drosophila cells: exploitation of early secretory apparatus dynamics. PLoS Pathog **2:**e34.
