# Supplementary material for: Host Cell S Phase Restricts Legionella pneumophila Intracellular Replication by Destabilizing the Membrane-Bound Replication Compartment
Source: mBio. 2017 Aug 22;8(4):e02345-16. doi: 10.1128/mBio.02345-16 (PMC5565972; doi:10.1128/mBio.02345-16)
Supplement: TABLE S2 [file mbo004173448st2.docx]

**Table S2. Percentage of wells showing a false positive**

| ***z* score** | **40** | **45** | **48** |
| --- | --- | --- | --- |
| **1** | 12% | 4% | 8% |
| **1.5** | 3% | 0% | 0% |
| **2** | 0% | 0% | 0% |

Strain Lp01 was used to challenge Drosophila Kc1167 cells seeded in 300 wells for the times noted (35-48 hrs). The ratios of areas of LCV/cell nuclei were determined for the 300 wells, and the standard deviation (sd) was determined to calculate *z* scores. Likelihood of selecting false mutants was obtained using the ratio of the number of wells present that were not within the indicated *z* scores. For instance, at 45hrs post infection, no control wells were detected more than 1.5 sd from the mean of the untreated wells.
