# Supplementary material for: Host Cell S Phase Restricts Legionella pneumophila Intracellular Replication by Destabilizing the Membrane-Bound Replication Compartment
Source: mBio. 2017 Aug 22;8(4):e02345-16. doi: 10.1128/mBio.02345-16 (PMC5565972; doi:10.1128/mBio.02345-16)
Supplement: FIG S1 [file mbo004173448sf1.pdf]

Supplemental Figure

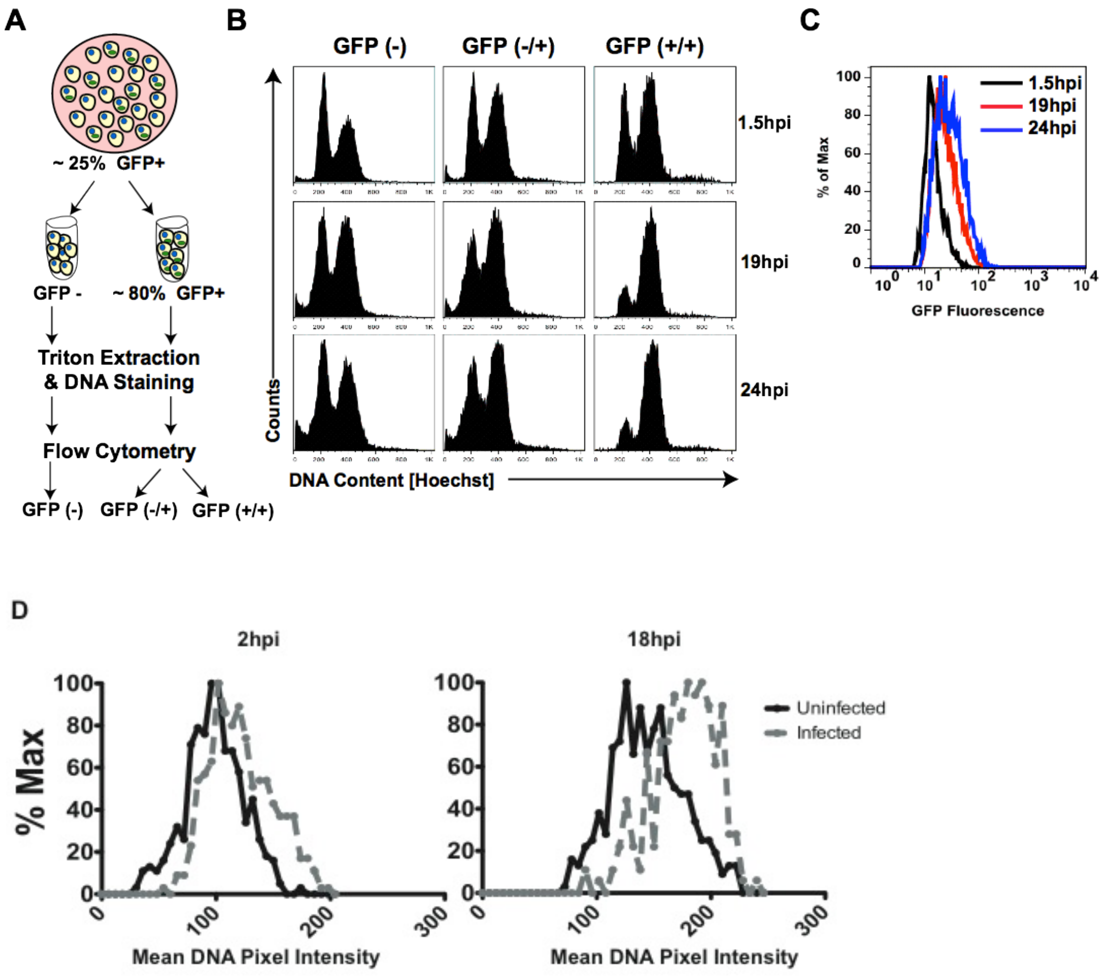

Figure S1. Cell cycle analyses of nuclei isolated from *Drosophila* cells challenged with *Legionella*-pGFP various times after uptake. *Drosophila* cells were challenged with *L. pneumophila* expressing GFP at MOI= 1 and at 2, 19 and 24hr after uptake. Infected cells were sorted from uninfected cells based on GFP fluorescence. Nuclei were isolated from infected and uninfected populations by Triton X100 extraction and stained with 1μg/ml Hoechst to determine DNA content distribution by flow cytometry. A) Schematic diagram of nuclei isolation process. B) DNA content distribution of isolated nuclei. C) GFP (+/+) nuclei were in tight association with phagosomes where replication had occurred. GFP(-/-) represents nuclei from uninfected cells that remained GFP -; GFP(+/-) are nuclei from infected cells that was GFP- after nuclei extraction; (GFP+/+) are nuclei from infected cells that was GFP+ after nuclei extraction. (D) At 2 and 18hpi infected cells were sorted from uninfected cells based on GFP fluorescence and seeded on 24-well plates containing coverslips. DNA was stained with Hoechst for GFP (-) and GFP (+) populations and analyzed microscopically.
