# Supplementary material for: Host Cell S Phase Restricts Legionella pneumophila Intracellular Replication by Destabilizing the Membrane-Bound Replication Compartment
Source: mBio. 2017 Aug 22;8(4):e02345-16. doi: 10.1128/mBio.02345-16 (PMC5565972; doi:10.1128/mBio.02345-16)
Supplement: TABLE S3 [file mbo004173448st3.docx]

**Table S3. Strains, Plasmids and Oligonucleotides**

**Strains**

***L. pneumophila***

| Strain | Genotype | Description | Reference |
| --- | --- | --- | --- |
| Lp01 | Philadelphia 1, *rpsL*, *hasdR* | wild-type strain | ([2](#_ENREF_2), [3](#_ENREF_3)) |
| Lp02 | Philadelphia 1, *thyA* *rpsL, hsdR* | wild-type strain | ([2](#_ENREF_2), [3](#_ENREF_3)) |
| Lp03 | *thyA* *rpsL* *hsdR dotA03* | T4SS translocation deficient | ([2](#_ENREF_2), [3](#_ENREF_3)) |
| Lp02*thyA*^+^ | Lp02 *thyA+* |  | this work |
| Lp03*thyA*^+^ | Lp03 *thyA+* |  | this work |

**Plasmids**

| Plasmid | Features | Description | Reference |
| --- | --- | --- | --- |
| pJB3395 | *thyA*+, amp^R^ | *thyA* allelic exchange vector | ([4](#_ENREF_4)) |
| pAM239 | GFP | GFP expression | ([5](#_ENREF_5)) |
| p*ahpC::lux* | *luxCDABE,* kan^R^ | luciferase expression | ([2](#_ENREF_2)) |

**Oligonucleotides**

| Name | Sequence |
| --- | --- |
| eIF2alpha-F | TAATACGACTCACTATAGGGACTTTAACATGGCCCTGACG |
| eIF2alpha-R | TAATACGACTCACTATAGGGCAGGCGGTCTTCTGGTAGAG |
| eIF3p40-F | TAATACGACTCACTATAGGGGTGCCTGGAGATCACCAACT |
| eIF3p40-R | TAATACGACTCACTATAGGGCGTTCAGCTCGCTCATCATA |
| RpL10Ab-F | TAATACGACTCACTATAGGGTGATACGCTGTATGAGGGCGTCAA |
| RpL10Ab-R | TAATACGACTCACTATAGGGTCTTGGGACGAGGAATGTGCTTCA |
| RpL27-F | TAATACGACTCACTATAGGGCTAAGCTGCCCATTCGCTACTTG |
| RpL27-R | TAATACGACTCACTATAGGGTTCGGGCGGTTAAGGCTATGGAAT |
| RpS30-F | TAATACGACTCACTATAGGGATCGATAATTCTGCGCCATC |
| RpS30-R | TAATACGACTCACTATAGGGTCTGGGCCAGTTGGTTCTAC |
| RpS7-F | TAATACGACTCACTATAGGGCGCAGTGCAGCGAAATAATA |
| RpS7-R | TAATACGACTCACTATAGGGTACCTTCTTGCTGCCGAACT |
| Ef1alpha100E-F | TAATACGACTCACTATAGGGAACATGTATCGTCACCGCAA |
| Ef1alpha100E-R | TAATACGACTCACTATAGGGGCAATGAGATGACATGTGGC |
| eIF1A-F | TAATACGACTCACTATAGGGGATTAACCAGGGCGACATCA |
| eIF1A-R | TAATACGACTCACTATAGGGAGACAGACGTTCCAGACGCT |
| Ef1alpha48D-F | TAATACGACTCACTATAGGGGAGTGCACACTGGGAAAACA |
| Ef1alpha48D-R | TAATACGACTCACTATAGGGGGAGGCAACAAGCAAAGAGA |
| Actin5-F | ATTCTGCCATTCCACACACA |
| Actin5-R | GAAACTGCAGCCAAGTGTGA |
| Oligo_dT | TTTTTTTTTTTTTTTTTTTTTTT |
| CycA-F | TAATACGACTCACTATAGGGCACGAACCGCTGAACAAGTA |
| CycA-R | TAATACGACTCACTATAGGGTTGCCTTCTTGCTGTTGTTG |
| eIF1A-F | TAATACGACTCACTATAGGGAGCGTCTGGAACGTCTGTCT |
| eIF1A-R | TAATACGACTCACTATAGGGCCATTTCTTGCACAGCGTAA |
| stgF1 | TAATACGACTCACTATAGGGGGAGGAGCTGTCGTTCTACG |
| stgR1 | TAATACGACTCACTATAGGGGGCAGTTCTCCTTCTCAACG |
| CycE-F1 | TAATACGACTCACTATAGGGGACCCTGAACTCGGTTTTGA |
| CycE-R1 | TAATACGACTCACTATAGGGTGTCGTCCAAAAGGTCATCA |
| Twine-F1 | TAATACGACTCACTATAGGGGCTCGATGTGGAGGAAGAAG |
| Twine-R1 | TAATACGACTCACTATAGGGGCTTTCCGATTTCAGCTGTC |
| Dup(Cdt1)-F1 | TAATACGACTCACTATAGGGTCAACGAAGACGATGTGCTC |
| Dup(Cdt1)-R1 | TAATACGACTCACTATAGGGGCTTGCTGTTTTTCCGACTC |
| eIF4G-F1 | TAATACGACTCACTATAGGGACTCACAACCCCTTCACCAG |
| eIF4G-R1 | TAATACGACTCACTATAGGGTTACACCAACCCCAACCATT |
| Geminin_F | TAATACGACTCACTATAGGGCGGATACCCAGACTGATGCT |
| Geminin_R | TAATACGACTCACTATAGGGCGTGTGATTGGTGTGTAGCC |
| RnrS_F | TAATACGACTCACTATAGGGGACCTTCTCCAACGAGCTTA |
| RnrS_R | TAATACGACTCACTATAGGGCATCCAGCGATATCATCTCC |
| eIF4E_F | TAATACGACTCACTATAGGGACACACGCACCAACCAACTA |
| eIF4E_R | TAATACGACTCACTATAGGGGAACATGGACTTGGGATTGG |
| eIF4E_F2 | TAATACGACTCACTATAGGGGTGGTGAGCGGATGGTCTAT |
| eIF4E_R2 | TAATACGACTCACTATAGGGGCAGCTTGTGACCAATCTCA |

**References**:

1. **Dorer MS, Kirton D, Bader JS, Isberg RR.** 2006. RNA interference analysis of Legionella in Drosophila cells: exploitation of early secretory apparatus dynamics. PLoS Pathog **2:**e34.

2. **Ensminger AW, Yassin Y, Miron A, Isberg RR.** 2012. Experimental evolution of Legionella pneumophila in mouse macrophages leads to strains with altered determinants of environmental survival. PLoS Pathog **8:**e1002731.

3. **Berger KH, Isberg RR.** 1993. Two distinct defects in intracellular growth complemented by a single genetic locus in Legionella pneumophila. Mol Microbiol **7:**7-19.

4. **Merriam JJ, Mathur R, Maxfield-Boumil R, Isberg RR.** 1997. Analysis of the Legionella pneumophila fliI gene: intracellular growth of a defined mutant defective for flagellum biosynthesis. Infect Immun **65:**2497-2501.

5. **Losick VP, Isberg RR.** 2006. NF-kappaB translocation prevents host cell death after low-dose challenge by Legionella pneumophila. J Exp Med **203:**2177-2189.
