# Supplementary material for: Host Cell S Phase Restricts Legionella pneumophila Intracellular Replication by Destabilizing the Membrane-Bound Replication Compartment
Source: mBio. 2017 Aug 22;8(4):e02345-16. doi: 10.1128/mBio.02345-16 (PMC5565972; doi:10.1128/mBio.02345-16)
Supplement: TABLE S4 [file mbo004173448st4.docx]

**Table S****4. Multiplicity of infection (MOI) used per assay**

| Cell type | MOI | Assay |
| --- | --- | --- |
| *Drosophila melanogaster* Kc167 | 1 | dsRNA Screen/Flow cytometry |
| *Drosophila melanogaster* Kc167 | 2 | Immunofluorescence |
| HeLa cells | 15 | Flow cytometry |
| HeLa cells | 10 | Immunofluorescence |
